# Corrosion of Carbon Steel in Artificial Geothermal Brine: Influence of Carbon Dioxide at 70 °C and 150 °C

**DOI:** 10.3390/ma12223801

**Published:** 2019-11-19

**Authors:** Gabriela Aristia, Le Quynh Hoa, Ralph Bäßler

**Affiliations:** 1 Department of Physical and Theoretical Chemistry, Freie Universität Berlin, 14195 Berlin, Germany; 2 Federal Institute for Materials Research and Testing (BAM), Unter den Eichen 87, 12205 Berlin, Germany; quynh-hoa.le@bam.de (L.Q.H.); ralph.baessler@bam.de (R.B.)

**Keywords:** carbon steel, CO_2_, corrosion, electrochemical impedance spectroscopy, geothermal

## Abstract

This study focuses on the corrosion mechanism of carbon steel exposed to an artificial geothermal brine influenced by carbon dioxide (CO_2_) gas. The tested brine simulates a geothermal source in Sibayak, Indonesia, containing 1500 mg/L of Cl^−^, 20 mg/L of SO_4_^2−^, and 15 mg/L of HCO_3_^−^ with pH 4. To reveal the temperature effect on the corrosion behavior of carbon steel, exposure and electrochemical tests were carried out at 70 °C and 150 °C. Surface analysis of corroded specimens showed localized corrosion at both temperatures, despite the formation of corrosion products on the surface. After 7 days at 150 °C, SEM images showed the formation of an adherent, dense, and crystalline FeCO_3_ layer. Whereas at 70 °C, the corrosion products consisted of chukanovite (Fe_2_(OH)_2_CO_3_) and siderite (FeCO_3_), which are less dense and less protective than that at 150 °C. Control experiments under Ar-environment were used to investigate the corrosive effect of CO_2_. Free corrosion potential (E_corr_) and electrochemical impedance spectroscopy (EIS) confirm that at both temperatures, the corrosive effect of CO_2_ was more significant compared to that measured in the Ar-containing solution. In terms of temperature effect, carbon steel remained active at 70 °C, while at 150 °C, it became passive due to the FeCO_3_ formation. These results suggest that carbon steel is more susceptible to corrosion at the near ground surface of a geothermal well, whereas at a deeper well with a higher temperature, there is a possible risk of scaling (FeCO_3_ layer). A longer exposure test at 150 °C with a stagnant solution for 28 days, however, showed the unstable FeCO_3_ layer and therefore a deeper localized corrosion compared to that of seven-day exposed specimens.

## 1. Introduction

Geothermal energy is an alternative to reducing the dependency on fossil fuels [1,2]. Although its future is promising, its implementation is facing various challenges. The first step of implementing geothermal energy is extracting the energy resource by pumping hot fluids from a geothermal well. Here, one of the major challenges is the corrosive nature of the geothermal fluid due to its composition based on different ionic species and gases under a wide range of temperatures [3,4,5]. Consequently, the infrastructure of geothermal powerplants may undergo corrosion and scaling when exposed to the geothermal fluid [6,7,8,9]. Therefore, it is necessary to assess the risks encountered during the operation and maintenance of geothermal powerplants to ensure its safety and structural integrity. Such a risk assessment will enhance the longevity of the powerplants and will consequently reduce the cost of energy production.

In terms of potentials in geothermal energy, Indonesia stands out with an estimated preserved energy of 29 GWe [10]. Most of the geothermal wells in Indonesia are located around the volcanic regions and usually contain non-condensable gases, e.g., carbon dioxide (CO_2_) and hydrogen sulfide (H_2_S) [11,12]. Geothermal wells in Sibayak (North Sumatera), Indonesia, belong to young stratovolcanoes with operating temperatures varying from 36 °C at the near ground surface to 310 °C at the bottom of the well [12,13]. According to the geothermal fluid classification [5,14], the Sibayak fluid is liquid dominated that has the corrosivity of type II with acidic and saline properties. This geothermal brine, therefore, creates an aggressive environment that is conducive to corrosion of the powerplant infrastructure, e.g., pipelines made of carbon steel.

Carbon steel is widely used for building geothermal powerplants because it offers good mechanical properties, machinability, and, weldability with a low cost [15]. To date, there have been several studies on the corrosion behavior of carbon steel in different geothermal fluids [9,16,17]. Klapper et al. [16] showed that carbon-steel grades API L80 and API Q125 exhibited an exceptional corrosion resistance and negligible susceptibility to localized corrosion. This study was performed at 100 °C and 150 °C using a deaerated artificial geothermal solution simulating the southern German Molasse Basin. On the other hand, Keserovic et al. showed that the carbon-steel grade API Q125 [17] is suitable for a stagnant geothermal brine. The study was performed at 175 °C and 900 kPa using an aerated geothermal solution simulating the Sibayak geothermal brine. These two works provide insight into carbon steel usage in geothermal brines; however, the testing conditions were limited to the aerated and deaerated solutions, which do not represent the real gaseous composition, i.e., CO_2_ containing atmosphere. Therefore, the influence of dry CO_2_ on the corrosion of carbon steel in the geothermal brine needs to be studied. 

In the presence of CO_2_ gas, carbon steel is more susceptible to corrosion as compared to those exposed to the deaerated solution [18]. Although CO_2_ alone is not hazardous to carbon steel, its interaction with water results in carbonic acid, which induces corrosion [19]. To understand the underlying mechanism, many studies have investigated the influence of CO_2_ on the corrosion of carbon steel using different concentrations of NaCl solution [20,21,22] and brines [23,24]. In 1 wt.% NaCl solution at pH 4–6.6 and CO_2_ partial pressure (P_CO2_) below 5 bar, there was no corrosion product layer below 60 °C [25]. However, Fe_2_(OH)_2_CO_3_ was found at about 60 °C in 3 wt.% NaCl solution with pH 5 [26], whereas FeCO_3_ and magnetite (Fe_3_O_4_) based corrosion products were formed between 130 °C–200 °C in 1 wt.% NaCl solution at pH 3.83 with P_CO2_ of 1 bar (prepared at room temperature) [27]. Pitting corrosion is usually also observed within 60 °C–130 °C where FeCO_3_ is not dominant. 

Despite the useful information of the temperature dependency on the corrosion products formation, there is an inconsistency in terms of the experimental parameters, i.e., concentration of NaCl, pH, and P_CO2_. The protectiveness of FeCO_3_ depends not only on temperature but also on different factors, such as pH, the microstructure of carbon steel, flowing condition, and CO_2_ partial pressure/concentration [20,22,25,28,29]. The fact that CO_2_ gas is present in several Sibayak wells with a stream of 80–90 mol.% [13], motivates our work to take into account the effect of CO_2_ on the corrosion of carbon steel. To the best of our knowledge, this is the first time that the effect of CO_2_ is included to study the corrosion of carbon steel in the Sibayak geothermal brine.

To study the influence of CO_2_ on the corrosion mechanism of carbon steel in geothermal brines, this work uses an artificial Sibayak geothermal brine at 70 °C and 150 °C with and without CO_2_ gas. Free corrosion potential and EIS were performed to trace the kinetics of the corrosion processes, followed by surface analysis to investigate the corrosion mechanism of carbon steel exposed to a CO_2_-containing brine.

## 2. Materials and Methods

### 2.1. Materials and Sample Preparation

The specimens used in this study is carbon steel St-37 (German name) or S 235 JR (European name). St-37 is ferritic steel (ThyssenKrupp, Essen, Germany), usually used for construction, with a tensile strength of 340–470 N/mm^2^. The elemental composition of carbon steel was characterized using spark-optical emission spectroscopy (SPECTRO Analytical Instruments GmbH, Kleve, Germany), as shown in Table 1. 

The specimens were prepared according to the ASTM G1-03 Standard Practice for Preparing, Cleaning, and Evaluating Corrosion Test Specimen [30]. For static exposure tests, three carbon steel specimens were used for each experiment. The specimens were manufactured with the dimension of 50 mm × 20 mm × 3 mm. Surfaces were ground using 80, 120, and 320-grit silicon carbide abrasive papers, and cleaned using bi-distilled water and degreased by acetone to have the same starting surface conditions for all experiments. For electrochemical tests, specimens have the dimension of 20 mm × 15 mm × 3 mm and were prepared with the same procedure, which then spot-welded to Ni-Cr based rod with a diameter of 2 mm.

### 2.2. Experimental Condition

Exposure and electrochemical tests were conducted in an artificial geothermal brine [17], based on a realistic composition of geothermal brine found in Sibayak, Indonesia [13], as shown in Table 2. 

Experiments were conducted at 70 °C and 150 °C. At 70 °C, exposure tests and electrochemical tests were performed under ambient pressure in a 500-mL glass vessel. The vessel was equipped with a temperature regulator and a temperature sensor Pt-100 (Thermocoax Isopad GmbH, Heidelberg, Germany) with a precision of ±3 °C. Prior to CO_2_ purging, the solution was purged by Ar for 30 min to remove oxygen. A continuous purging of CO_2_ gas was applied throughout exposure time. To reveal the effect of CO_2_, control experiments without CO_2_ were performed, in which Ar was purged continuously. 

To perform the experiments at 150 °C, 500 mL of solution was purged by Ar for 30 min and followed by CO_2_ for 30 min. The experiments were performed in the autoclaves with CO_2_ partial pressure of 500 kPa applied at room temperature, achieving a total pressure of 1 MPa at 150 °C. Because the CO_2_ gas was purged before starting the experiment instead of using a continuous flow for seven days, the solution condition is hereafter mentioned as a CO_2_-containing solution. For control experiments, Ar was purged in the solution for 30 min and applied to the autoclave with Ar partial pressure of 500 kPa at room temperature. To avoid contamination and electrical interferences between the autoclave and the electrochemical system, a glass vessel was placed in the autoclave. For the exposure test, autoclaves were kept undisturbed in a climate chamber at 150 °C. The electrochemical test was performed in autoclaves at 150 °C with the same initial condition as the exposure test, albeit the temperature was maintained at 150 °C by using a heating plate. 

### 2.3. Methods

#### 2.3.1. Exposure Test

Three specimens were exposed for seven days, at 25 °C, 70 °C, and 150 °C. To investigate the effect of gaseous condition on the corrosion of carbon steel in artificial geothermal brines, aerated, deaerated, and CO_2_-containing solutions were used. Exposure tests at 150 °C were performed further for 28 days to investigate the time effect on the corrosion behavior of carbon steel in deaerated and CO_2_-containing solution. Corrosion rate was determined based on gravimetry, as outlined in ASTM G1-03. Specimens were weighed before and after exposure tests to calculate the corrosion rate.

#### 2.3.2. Surface Characterization

Post-experimental analyses include macro-photography and surface morphological characterization using a Scanning Electron Microscope (SEM) of exposed specimens. Chemical analysis was performed by using Energy Dispersive X-Ray (EDX), Grazing Incidence X-ray Diffraction (GI-XRD), and Electron Backscatter Diffraction (EBSD) to identify the elemental composition, their distribution, and the specific phases of corrosion products related to the morphology observed by SEM. GI-XRD was used as a surface sensitive method to detect the corrosion product directly on the specimen surface, with 2θ from 10°–70°, resolution of 0.02°, and X-ray source of Cobalt K-α. Results of XRD data was converted to the Cu-Kα source and analyzed using Match! Crystal Impact software and the converted data was matched to reference XRD data contained in the Crystallography Open Database (COD). Cross sections were prepared by embedding the specimens in epoxy resins, then cutting and polishing to observe the possible localized corrosion as well as to determine the thickness of the corrosion products. 

#### 2.3.3. Electrochemical Test

A standard three-electrode system was used in the electrochemical measurements, with a Ag/AgCl-saturated reference electrode and a Ti/TiO_2_ mesh counter electrode. For all experiments, open circuit potential (OCP), also known as free corrosion potential (E_corr_), was measured for seven days to investigate the spontaneous corrosion behavior. Electrochemical data were immediately recorded after the desired temperature was reached.

Electrochemical Impedance Spectroscopy (EIS) was applied with an interval of 2 h for the first 12 h to investigate the early reacting stage. After the first day, EIS was performed with an interval of 24 h. EIS data were recorded within a frequency range of 10^4^–10^−1^ Hz for the first 12 h and 10^4^–10^−2^ Hz after 24 h. The shorter frequency range in the first few hours was used to avoid unstable data. All EIS data were collected using 10 points/decade, with an amplitude of 10 mV vs. E_corr_. 

Electrochemical data were recorded by Gamry Reference 600 potentiostat (Gamry Instruments, Inc., Warminster, PA, USA) and analyzed using Gamry Echem Analyst software (Gamry Instruments, Inc., Warminster, PA, USA). Comparative studies of OCP and EIS were conducted for Ar and CO_2_-containing solutions. Electrochemical impedance data were presented in the Nyquist plot, Bode plot, and Phase angle plot. Nyquist plot shows the evolution of impedance, in both real (Z_real_) and imaginary (Z_imag_) part, over time. Bode Plot presents the total impedance |Z| versus frequency. Phase angle plot was used to observe the phase angle with respect to frequency. Electrochemical impedance spectra were further fitted to electrochemical equivalent circuits to find out the corresponding corrosion behavior of the product layer over time.

## 3. Results and Discussion

### 3.1. Effect of Temperature and CO_2_ on the Corrosion Rate

**Figure 1:**
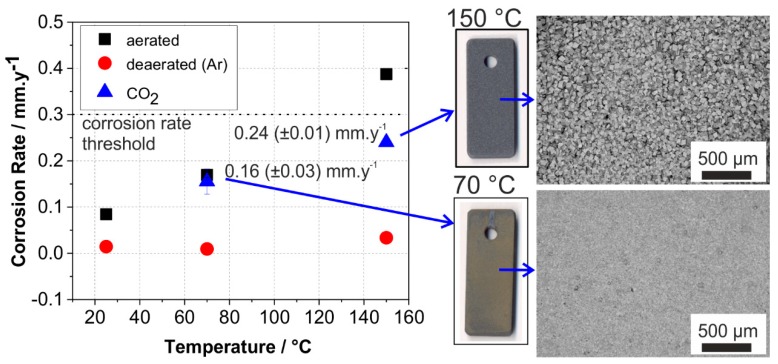
Corrosion rates after seven days of different exposure tests and the corresponding macro-photos and Scanning Electron Microscope (SEM) images of the tested carbon steel specimens exposed at 70 °C and 150 °C.

To have a general overview on the effect of solution solely (without gases) and with different gases on the corrosion behaviors of carbon steel, exposure tests were performed for seven days in three conditions: deaerated with Ar, aerated, and CO_2_-containing solutions at 25, 70 and 150 °C. Figure 1 shows the corrosion rate of carbon steel as a function of temperature after seven-day exposure tests. For industrial application, the corrosion rate threshold is usually set to 0.3 mm/year to ensure the safety of material constructions, albeit with the requirement that there is no localized or pitting corrosion observed [7]. As shown in the results of deaerated tests, although the solution contains 1500 mg/L chloride, Sibayak solution alone did not lead to a corrosion rate of more than 0.1 mm/year, regardless the testing temperatures. When dissolved oxygen is present, as simulated by the aerated solution, the corrosion rate significantly increases with temperature, suggesting that dissolved oxygen contributes to accelerating corrosion reaction. This result is in agreement with previous studies and confirms the presence of oxygen to be the main cause of corrosion rate increase [9,31,32]. In the geothermal water medium, 30 ppb of oxygen causes up to four times increase of corrosion rate on carbon steel, while the presence of oxygen with concentrations higher than 50 ppb causes serious pitting corrosion [32,33]. Although dissolved oxygen decreases with the increase of temperature, there is a possibility that oxygen traces intrude to a certain depth of a geothermal well and cause corrosion [3,34,35]. 

Beside oxygen that may exist with a low concentration, CO_2_ presents in Sibayak geothermal system as dissolved gas with a stream of 80–90 mol.% [13]. Basing on the results of weight loss determination, under the presence of CO_2_ at 70 °C and 150 °C, corrosion rates were 0.16 and 0.24 mm/year, respectively. Similar to the corrosion rates of carbon steel in the presence of oxygen, corrosion rates of CO_2_ exposed specimens increase with temperature, but somewhat with a lower degree than in an oxygen-containing environment. The macro-photos and SEM images of tested specimens in the CO_2_ environment (Figure 1) show a very homogeneous and dense layer of corrosion products indicating uniform corrosion. For long-term exposure, the nature of corrosion products governs the continuity of corrosion processes. A resistive and dense layer of highly crystalline corrosion products will act as a protective barrier, while a porous layer might allow selective ions to penetrate through the metal surface, causing further corrosion processes. Therefore, it is necessary to investigate to what extent CO_2_ does influence the corrosion of carbon steel and the nature of its corrosion products.

### 3.2. Effect of Temperature on Corrosion Product Formation

**Figure 2:**
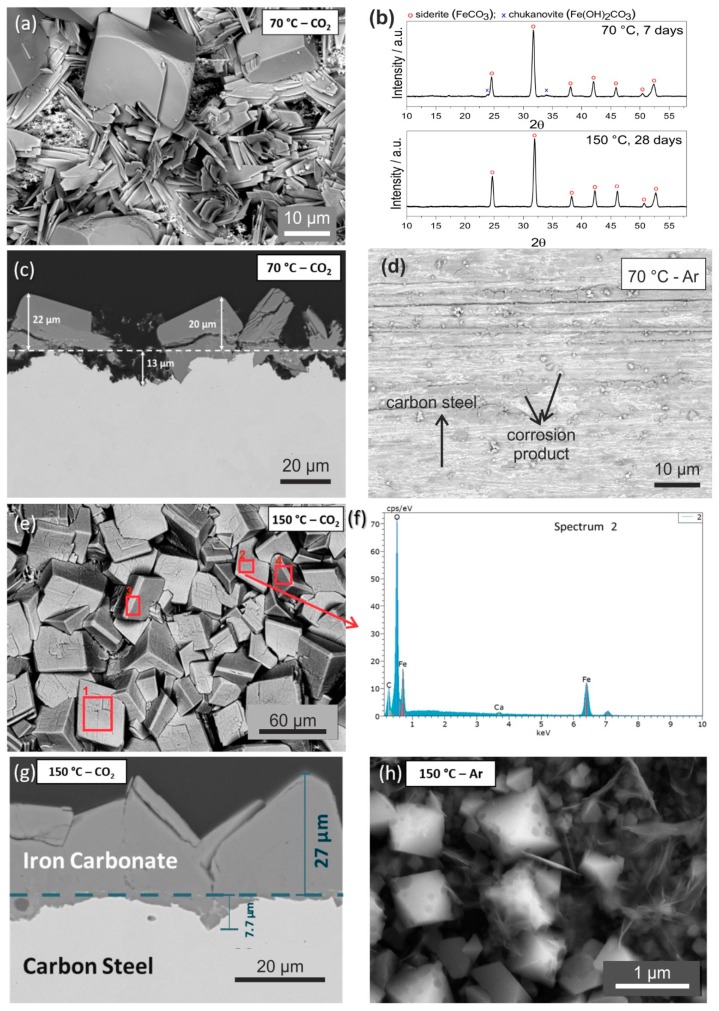
Characterization of carbon steel specimens after exposure to the artificial geothermal solution for seven days at 70 °C (**a**–**d**) compared to that at 150 °C (**e**–**h**). Experiments were performed with CO_2_ (**a**–**c**;**f**–**g**) and with Ar (**d**,**h**). (**b**) is the corresponding GI-XRD spectra of (**a**). (**f**) is the corresponding EDX spectrum of (**e**).

To identify the morphology and characteristics of the main corrosion products formed on the carbon steel surface, SEM images of carbon steel surface were taken. Figure 2 shows a higher magnification of SEM images after the carbon steel was exposed to 70 °C and 150 °C in CO_2_ and Ar condition for seven days. In conjunction with the surface morphology images, additional information is provided, such as the cross-section, elemental composition, and phases, as shown in Figure 2. 

In the CO_2_-saturated condition at 70 °C (Figure 2a), there are two dominant crystal structures, plate-like and prismatic-shaped crystals. X-ray diffraction data (Figure 2b) showed that the corrosion products were predominantly Fe_2_(OH)_2_CO_3_ chukanovite (COD 9010838) and FeCO_3_ siderite (COD 9000098). The plate-like crystal structure may be then assigned to chukanovite, whereas the prismatic cubic shape crystal is a characteristic of siderite crystal structure, as also reported in other studies [36,37,38]. Chukanovite exists as a metastable phase, commonly found in a laboratory scale experiment instead of in the realistic field condition. This corrosion product does not adhere to the carbon steel surface, which leads to poor protectiveness. In CO_2_-saturated solution at 70 °C, siderite has a thickness of 10–22 µm, and chukanovite has a thickness of 5–18 µm. Underneath the corrosion product layer, localized corrosion was found with the depth of 6–15 µm (Figure 2c). As can be seen in Figure 2a, chukanovite was mixed with siderite and did not completely cover the carbon steel surface. Consequently, the resulting space between those two types of crystal structure opened pathways for the electrolyte to further react with the carbon steel surface (Figure 2c). Therefore, carbon steel in this condition is susceptible to a pit initiation. Without CO_2_, as represented by experiments under Ar, the thickness of the corrosion product layer is significantly reduced, and the products did not cover the entire surface (Figure 2d). The brighter area in the SEM image shows the iron surface that is not fully covered by the corrosion product. This result shows that at 70 °C more accumulation of corrosion product was observed in the CO_2_-containing solution (Figure 2a) than in the deaerated solution (Figure 2d), which confirms the corrosive effect of CO_2_ toward carbon steel in geothermal solution (Figure 1).

At 150 °C, the prismatic structure was observed as the dominant corrosion product (Figure 2e) identified as FeCO_3_. The appearance of a very homogeneous structure, hence the absence of the plate-like crystal structure, which was previously identified as chukanovite, showed that siderite formation is more favorable at 150 °C. Similar observations were also confirmed by other studies [22,39]. Saturation degree (S) is a key parameter in the formation and precipitation of corrosion products, depending on the ionic concentration (c) and solubility limit (K_sp_). For example, for FeCO_3_:

(1) $$S_{FeCO_{3}}=\;\frac{c_{Fe^{2+}}c_{CO_{3}^{2-}}}{K_{sp\;FeCO_{3}}}$$

When the concentration of Fe^2+^ and CO_3_^2−^ increases, more FeCO_3_ precipitates and crystalizes on the surface. At 70 °C, FeCO_3_ was formed; however, this temperature is not high enough to form the dense and protective FeCO_3_. Thus, the experimental results evidently showed that the precipitation and formation of FeCO_3_ are highly favored at a higher temperature. 

To confirm the FeCO_3_ formation on the carbon steel surface at 150 °C, EDX was used to identify the constituent elements of the corrosion product. Several points were selected and analysis by EDX (Figure 2f) revealed not only Fe, C, and O, but also a small amount of Ca. The detected Ca element can be associated with the incorporation of Ca^2+^ ion into the FeCO_3_ structure, due to the isostructural behavior of CaCO_3_ and FeCO_3_ [40]. EDX results showed the atomic ratio of Ca and Fe, leading to x = 0.98 (±0.01) and y = 0.02 (±0.01) for a mixed carbonate compound Fe_x_Ca_y_CO_3_. In an extended exposure test, the EDX spectra showed that the atomic ratio between Ca and Fe changed, resulting in x = 0.96 and y = 0.04. This change indicated that Ca^2+^ can further diffuse to the FeCO_3_ structure when the specimen is exposed with a longer time.

The incorporation of Ca^2+^ in this experiment (at 150 °C, with 200 ppm Ca^2+^) is considerably low compared to another study where 100 ppm Ca^2+^ was used in stagnant condition at 80 °C, resulting in a y value of 0.22 [41]. It is also noteworthy to mention that different brine chemistry affects the incorporation of Ca^2+^ to the FeCO_3_ structure, as the experiment was not performed in a pure CaCl_2_ solution, but rather in an artificial geothermal brine having various ionic species. Additionally, this study uses higher temperature and stagnant solution at 150 °C. As concluded by Mansoori et al. [40], it is still debatable whether Ca^2+^ has a positive or negative influence on the corrosion behavior of different brine chemistry and experimental conditions. 

At 150 °C, a thickness of the corrosion product layer was measured between 9–27 µm (Figure 2g). There was also a take-up of carbon steel of 5–8 µm, which may be associated with the iron dissolution in the initial stage of exposure, followed by FeCO_3_ formation. Without CO_2_ gas, corrosion products have very small crystal size around 1 µm (Figure 2h), which is 60 times smaller than that observed in the CO_2_ environment (Figure 2e), which indicate that the type of dissolved gas influences the corrosion processes. 

**Figure 3:**
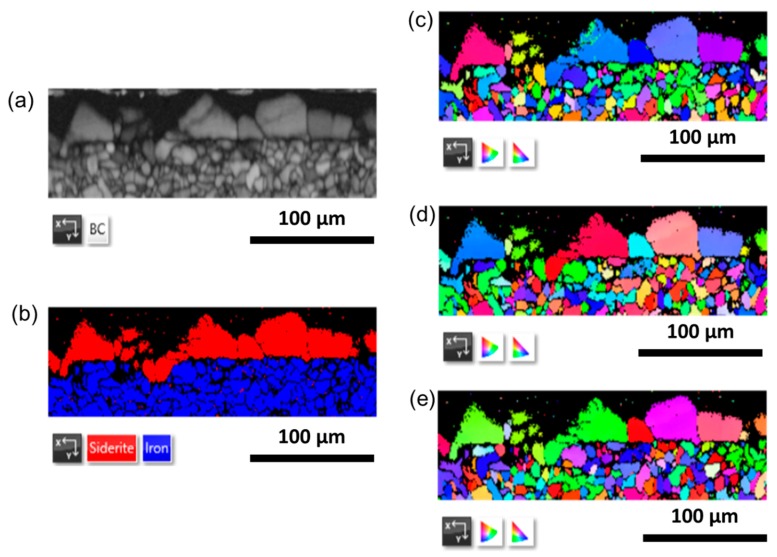
Electron Backscatter Diffraction (EBSD) mapping of the carbon steel cross-section specimen after exposure to the artificial geothermal solution for seven days at 150 °C: (**a**) pattern quality (**b**) phase composition (**c**–**e**) crystal orientation map, with inverse pole figure (IPF) coloring of x, y, and z direction.

As suggested by Tanupabrungsun et al. [27], there is a possibility that at 150 °C FeCO_3_ and magnetite (Fe_3_O_4_) are formed simultaneously. This phenomenon was simulated by a Pourbaix diagram of a Fe-CO_2_-H_2_O system at 150 °C with 10 ppm Fe^2+^ and 10 ppm Fe^3+^, which was also proven by an experimental work. Given this possibility, EBSD analysis was performed to ensure the composition of the corrosion products more precisely. Figure 3 shows the EBSD mapping of the carbon steel cross section, in which carbon steel was exposed to the CO_2_-containing geothermal water at 150 °C for seven days.

Based on the EBSD mapping, the phase composition of the cross section consists of siderite on the top layer and iron on the bottom layer as the base material (Figure 3b). This result confirmed that in this experimental condition, FeCO_3_ was formed at 150 °C without the concurrence of Fe_3_O_4_ formation. In addition, the crystal orientation map shows that the grain size of siderite varies from about 10–60 µm (Figure 3c–e), which is about similar to the crystal size of FeCO_3_, as observed from the top view of the specimen using SEM (Figure 2e).

**Figure 4:**
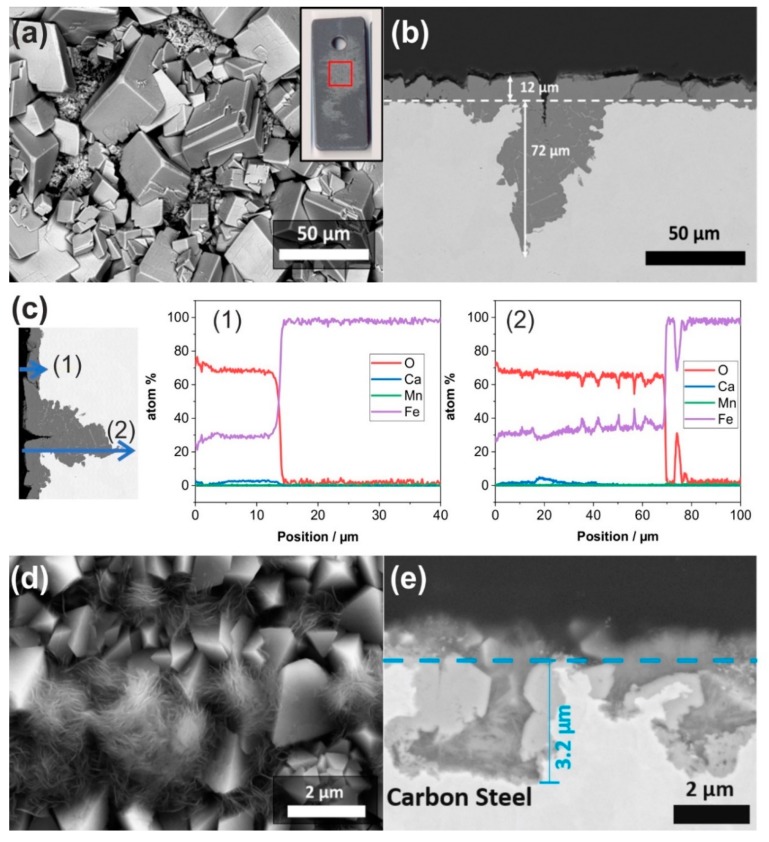
SEM images of the carbon steel specimen after exposure to the artificial geothermal solution for 28 days at 150 °C with CO_2_ (**a**–**c**) compared to that with Ar (**d**,**e**). SEM images show the surface morphology (**a**,**d**) and cross-section (**b**,**e**). Elemental distribution of (**b**) is shown by EDX line scan (**c**), carbon is not presented due to the sample preparation using carbon sputtering.

Longer exposure tests were performed for 28 days to investigate the growth and stability of the corrosion product. After 28 days of exposure, surface inhomogeneity was observed by macro-photography (inserted in Figure 4a). The brighter area had a different morphology than that of the seven-day exposed specimen, whereas the darker area consisted of FeCO_3_ with an identical structure as Figure 2e. Within the brighter area, the corrosion products had smoother crystal, and there are some smaller prismatic structures (Figure 4a). Although the corrosion product was protective, the cross-section revealed localized pits of up to 72 µm (Figure 4b) with a reduction of the FeCO_3_ thickness to the range of 6–13 µm, compared to that of the seven-day exposed specimens. EDX line scanning of the specimen cross-section revealed the distribution of elements within the layer and the pit presented by line scan 1 and line scan 2, respectively (Figure 4c). Here, carbon composition is not shown due to the sample preparation using carbon sputtering. Ca was detected in the entire layer thickness (line scan 1) for about 12 µm, whereas within the pit, Ca was diminished after about 40 µm depth (line scan 2). Thus, it can be concluded that the diffusion of Ca is limited to a certain depth.

Figure 4d shows that in deaerated solution, carbon steel was entirely covered by a homogeneous and dense layer of corrosion products consisting of two distinct morphologies, i.e., prismatic and needle-like structure. When compared to the carbon steel surface after seven days of exposure (Figure 2h), the crystal structure has a more dense and bigger structure, indicating a continuous growth along exposure time. Although having a denser crystal structure, the corrosion product layer was not protective, where several pits were observed with pit depth of 3 µm after 28 days of exposure (Figure 4e). Pitting corrosion was not only observed in the presence of CO_2_ but also in the absence of CO_2_; however, to a less degree than when CO_2_ is present in the system. 

### 3.3. Effect of CO_2_ on the Corrosion Behavior

To study the effect of CO_2_ on the corrosion behavior of carbon steel in geothermal brine, electrochemical measurements, including open circuit potential and impedance spectroscopy, were performed both in a deaerated solution and a CO_2_-containing solution. 

**Figure 5:**
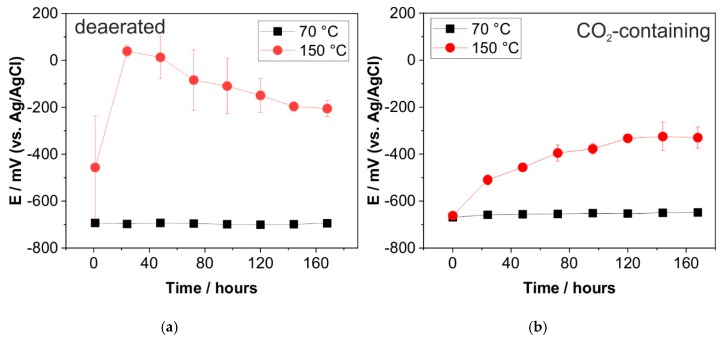
Open circuit potential of carbon steel in the artificial geothermal solution at 70 °C and 150 °C; (**a**) deaerated solution (**b**) CO_2_-containing solution.

Open circuit potential was recorded during exposure time to trace the corrosion reactions. The results were then selected at 24 h interval and plotted as E_corr_ versus time, as shown in Figure 5. At 70 °C, E_corr_ was between −693 to −701 mV for the specimen exposed to the deaerated solution (Figure 5a). E_corr_ was stable during the entire exposure, giving a maximum deviation of 8 mV, indicating a constant interaction of carbon steel surface with the brine without a significant change of the electrochemical reaction. As shown in Figure 2d, only a small amount of corrosion product is accumulated on the carbon steel, which then allows the brine to react directly with the metal surface, therefore it remains actively corroded. Similarly, E_corr_ of the specimens exposed to CO_2_-containing solution exhibited a small increase from −669 mV to −648 mV along the seven days of exposure. The most significant increase in E_corr_ was observed within 24 h, where the potential increased by 11 mV, suggesting a fast-initial dissolution of Fe^2+^ from the metal surface, followed by almost unchanged formation kinetics of the corrosion products. 

Electrochemical tests were then performed at 150 °C to reveal the effect of temperature on the corrosion process. Without CO_2_, there was a significant increase of E_corr_ after 24 h of exposure, from -457 mV to 39 mV, which then gradually decreased, reaching −206 mV after seven days. This result showed that in the first 24 h, a more cathodic reaction took place between Fe^2+^ and H_2_O, influenced by high applied temperature [42]. The gradual decrease of E_corr_ indicates a continuous active corrosion process after 24 h, albeit with a less degree compared to that at 70 °C. 

In the CO_2_-containing solution, E_corr_ increased significantly compared to the E_corr_ observed at 70 °C with CO_2_ gas, although not as significantly as at 150 °C in the deaerated solution in the first 24 h. The E_corr_ increase can be divided into two steps. The first increase was within the first 24 h, where the E_corr_ increased from −662 mV to −510 mV, giving an increase of 152 mV. The second increase of potential was with a slower rate, reaching saturation of E_corr_ on the fifth day of exposure (−335 mV), which corresponds to the growth of FeCO_3_ layer. As shown in Figure 4b, there was no significant difference in the potential between the fifth and seventh days of experiments. at 150 °C; unlike the OCP observed in the deaerated solution, E_corr_ increases gradually under the CO_2_ condition, indicating the formation of a passive layer and contributing to a more cathodic potential. 

**Figure 6:**
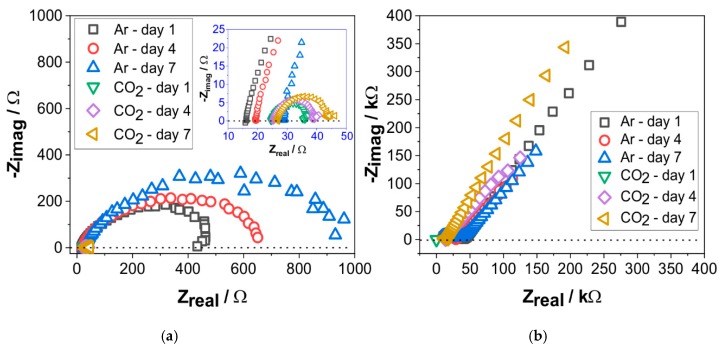
Electrochemical impedance spectra of carbon steel specimens exposed to deaerated (Ar) and CO_2_-containing geothermal solution (**a**) at 70 °C and (**b**) 150 °C.

Further investigation was performed by EIS to characterize the surface-interface interaction of carbon steel, electrolyte, and the corrosion products. Impedance data are presented in different formats to distinguish different specific behaviors. Complex-impedance-plane representation, usually also known by Nyquist plot, indicate the possible mechanism by the shape of the position of the points at each frequency. However, it does not explicitly show information about frequency; hence, the Bode Plot and Phase Angle plot provides more detailed information. Bode representation depicts the absolute impedance value with respect to frequency, whereas the Phase Angle plot shows the phase angle with respect to frequency. Figure 6 shows a comparison of the Nyquist plots from the exposed specimen between the deaerated and CO_2_-containing solutions. For both conditions, the Nyquist plot showed results on the negative value of Z_imag_ (y-axis), indicating a contribution of a capacitive reactance to the impedance value. 

At 70 °C, the impedance of specimen exposed to the deaerated solution exhibits a higher corrosion resistance than in the CO_2_-containing solution, indicated by a bigger diameter of the capacitive semicircles of more than 20 times (Figure 6a). Overall, the diameter of capacitive semicircle observed in both the deaerated and CO_2_-containing solution significantly increased over time, indicating a higher charge transfer resistance (R_ct_) over time, and therefore slower reaction kinetics on the surface. After seven days, the diameter of a capacitive semicircle observed in the deaerated solution was almost 50 times higher than in CO_2_-containing solution. By evaluating the change of Nyquist plot (Figure 6a) with an interval of three days, a significant increase was observed of almost 200 Ω in the deaerated solution and around 5 Ω in the CO_2_-containing solution. Although E_corr_ at 70 °C did not directly show the influence of CO_2_ (Figure 5), the EIS data evidently showed that carbon steel is more prone to corrosion in the CO_2_-containing solution than in the deaerated solution. 

**Figure 7:**
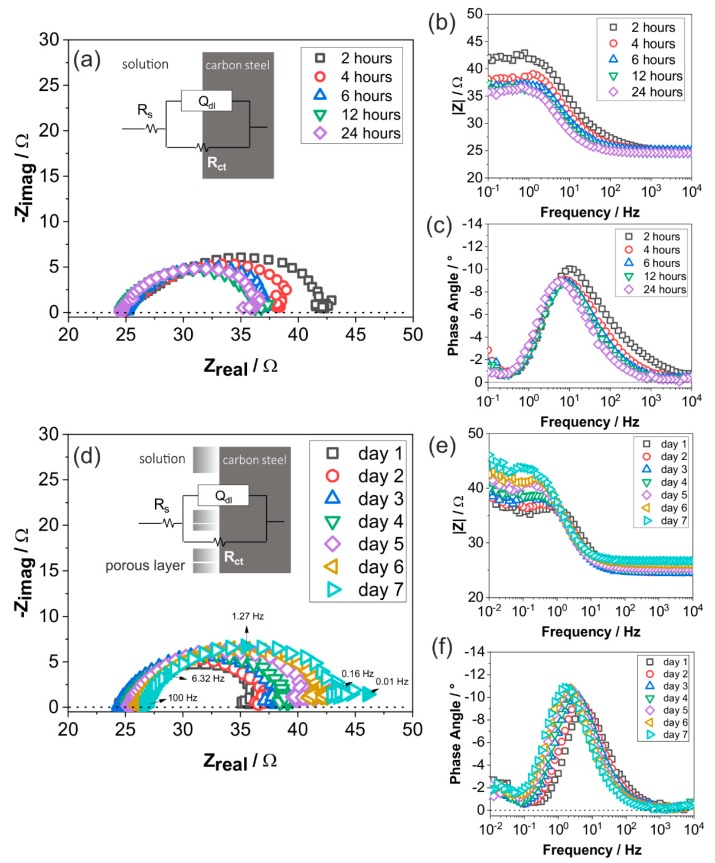
Electrochemical impedance spectra of carbon steel at 70 °C in the artificial geothermal solution with CO_2_ from 2–24 h presented in (**a**) Nyquist plot (**b**) Bode magnitude plot (**c**) Bode phase angle plot; and 1–7 days presented in (**d**) Nyquist plot (**e**) Bode magnitude plot (**f**) Bode phase angle plot.

**Figure 8:**
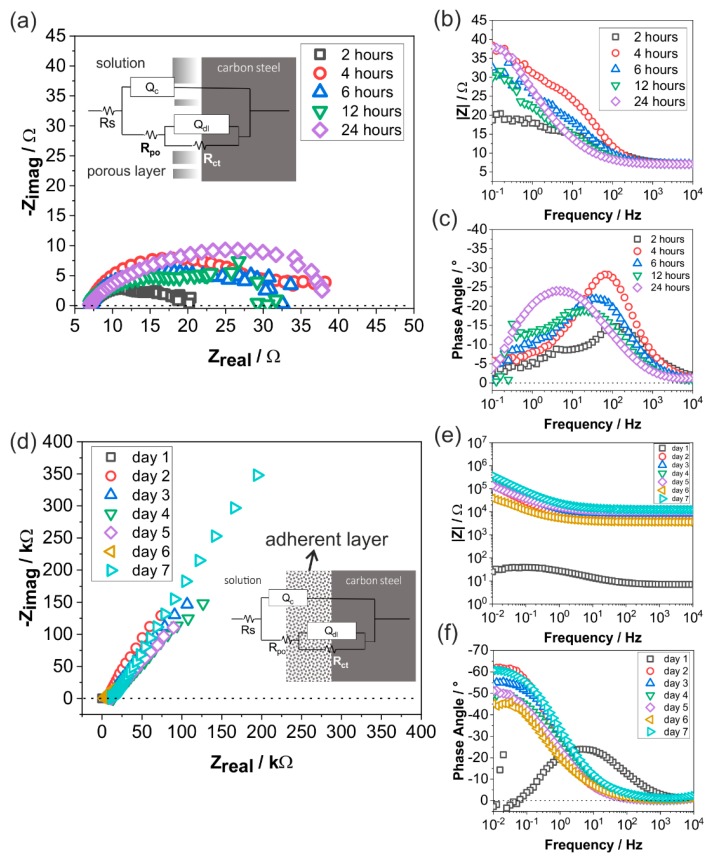
Electrochemical impedance spectra of carbon steel at 150 °C in the artificial geothermal solution with CO_2_ from 2 to 24 h presented in (**a**) Nyquist plot (**b**) Bode magnitude plot (**c**) Bode phase angle plot; and from the first to seventh day presented in (**d**) Nyquist plot (**e**) Bode magnitude plot (**f**) Bode phase angle plot.

The Nyquist plot showed an exponentially increased impedance of specimens at 150 °C (Figure 6b), compared to that at 70 °C (Figure 6a). Despite the higher corrosion rate of specimen exposed to the CO_2_-containing solution compared to that exposed to the deaerated solution at 150 °C (Figure 1), the Nyquist plot showed an indistinct high corrosion resistance of carbon steel in both solutions (Figure 6b). Instead of a capacitive semicircle, the Nyquist plot showed a diagonal line with a positive slope. Due to the limited frequency range, it is difficult to verify whether this pattern belongs to a much bigger capacitive semicircle, or is solely based on diffusion and mixed kinetic processes. To further interpret the data, additional information was analyzed based on the Bode plot, which has an advantage of presenting the total magnitude of impedance value and the phase angle as a function of frequency (Figure 7 and Figure 8).

### 3.4. Scaling Formation and Growth at Different Temperatures in a CO_2_ Environment

As revealed by OCP (Figure 5) and EIS data (Figure 6) in the previous section, the interdependency of free corrosion potential (OCP) and physical-electrical behavior (EIS) is not always straightforward to be interpreted. For example, Figure 5 showed an almost unchanging OCP of carbon steel at 70 °C, whereas Figure 6a showed a significant increase in the impedance of the corresponding specimen. As also discussed in the corrosion potential monitoring, the most important spontaneous reaction at 150 °C occurred within the first day (Figure 5), which necessitates a more precise EIS analysis within this initial stage to further analyze the kinetics of iron dissolution and the corrosion product formation. Thus, EIS data was recorded every 2 h in the first 12 h of exposure in addition to the day-to-day measurement. 

Figure 7 presents the evolution of impedance spectra in all three types of plots—Nyquist, Bode and phase angle plots—for each dataset, where carbon steel is exposed to the CO_2_ containing solution at 70 °C for seven days. 

Although there was only a very small change in free corrosion potential of carbon steel at 70 °C (Figure 5b), impedance spectra did show changes with respect to time. Within seven days of exposure, impedance spectra showed that the capacitive semicircles had diameter of less than 20 Ω, indicating a fast charge-transfer process on the surface of the tested specimen at 70 °C due to reactions with the CO_2_-containing artificial brine solution. Regardless of the stable impedance value over seven days, the impedance spectra did show two main stages of change: a decrease of impedance in the first day (Figure 7a–c); and a continuous, but slow increase of impedance from the first to seventh day (Figure 7d–f). 

During the first 24 h, impedance decreases, as shown in Figure 7a,b. Phase angle remained at around −9° at a high-to-medium frequency range, indicating that the characteristics of the charge transfer process are not capacitive, mainly because of metal dissolution. It is suggested that, on the first day, the fresh ground metal surface reacted with the solution, where selective dissolution of ferrite took place, and Fe was dissolved along with hydrogen evolution [21]. Low carbon steel is dominated by ferrite microstructure (α-Fe) with a secondary microstructure of cementite (Fe_3_C). As the ferrite dissolved, cementite remained on the surface, providing preferential cathodic sites with lower overpotential favoring hydrogen evolution [43]. This process resulted in the increment of charge-transfer processes and therefore the decrease of the characteristic semicircle diameter. In a similar test using carbon steel C1018 and deaerated 3% NaCl with pH 6 at 80 °C, Farelas et al. found a decrease of capacitive semicircle diameter until 15 h of exposure [21], which is comparable to this work, where a decrease of semicircle was also observed until 12 h of exposure. 

From the second day onwards (Figure 7d), the diameter of capacitive semicircles increased with respect to time. The dissolved Fe^2+^ formed corrosion products on the surface, as revealed by SEM image after seven days of exposure (Figure 2a). Although it covered the surface, the corrosion product was not strongly attached, allowing the metal to actively corrode, and therefore resulting in only a small increase of impedance. At frequency 10^−1^ to 10^−2^ Hz, Figure 7e shows that the magnitude of impedance slightly increased. The peak of the phase angle changed to a more negative value, indicating an increase of capacitance, which might be associated with the increase of surface coverage by the corrosion product. Consequently, the peak of phase angle shifted to a lower frequency, indicating slower kinetics of metal dissolution with respect to time. The increase of surface coverage by the corrosion product might hinder the mass transport towards the surface. Thus, the overall impedance was the highest by the end of the seventh day, indicating a higher corrosion resistance, although not to a significant extent.

At the lower frequency range, the Nyquist plot showed a small loop that took place both in the early stage of the first day (Figure 7a) and the later stage (Figure 7d). An inductive loop at low frequency is often observed, where CO_2_ was used in 3 wt.% NaCl solution [21], associated with reactive ionic species or adsorption of intermediate products. However, in this case, the loop was neither a distinct second semicircle (capacitive loop) nor an inductive loop at y-axis below 0 Ω (dotted line), which rather suggested an additional capacitive effect, instead of a simple inductive loop interpretation. 

A capacitive and/or inductive loop may deviate when an intermediate reaction occurs, e.g., dielectric relaxation, thickness modulation in the porous layer, and frequency-dependent impedance [44,45]. Frequency-dependent impedance is related to capacitive behavior, since a capacitor can charge and discharge at different frequency. One possible reason of capacitive and inductive loop deviation is the reaction between metal surface and the complex tested solution, i.e., 1500 mg/L of Cl^−^, 20 mg/L of SO_4_^2−^, and 15 mg/L of HCO_3_^−^. The role of Cl^−^ and SO_4_^2−^ on the iron dissolution mechanism has been studied by Barcia et al. [46], and it was shown that at pH 3 and 4 with an anodic polarization of 0.05 A/cm^2^, inductive loop in the lower frequency changes to capacitive loop. The same behavior was also observed by Bechet et al. [47] in sulfuric acid medium with pH 2.6 and anodic polarization of 100 mA/cm^2^. As HCO_3_^−^ is also present due to the deprotonation of hydrated CO_2_, having HCO_3_^−^ in the solution does not seem to have a significant influence on the reaction. 

As shown in Figure 5 and Figure 6b, there were significant changes not only in the E_corr_ but also in the impedance of specimens exposed at 150 °C, as compared to that at 70 °C, indicating a strong influence of temperature on the corrosion behavior. Similar measurement interval as that at 70 °C was applied at 150 °C to observe the early stage reaction within 24 h. The Nyquist plot of carbon steel exposed to 150 °C during the first few hours (2–24 h) revealed that electrolyte resistance was around 3.5 times lower than at 70 °C, i.e., 7 Ω, due to the increased temperature, resulting in a higher ionic mobility (Figure 8a). 

Between 2–12 h, the Nyquist plot does not present a single semicircle as that observed at 70 °C, but it does not distinctively show the second semicircle, resulting in the complexity in interpreting the exact underlying mechanism of corrosion in the first few hours. The Bode magnitude plot shows that there was an increase in absolute impedance value between 2–4 h, which then slightly decreased until 12 h, and then again increased at 24 h. Similar to that at 70 °C, there are an indication of two time-constants between 0–12 h (Figure 8c), albeit with a more complex phase angle graph with a second time constant (5 Hz–0.1 Hz) that cannot be interpreted assuredly. Compared to 70 °C, EIS data (Figure 8a–c) showed more varying patterns within 24 h, indicating more complex, unstable, and rapidly evolved processes. 

Figure 8d shows a Nyquist plot between 1–7 days, where a significant increase of impedance was observed in comparison with the first 24 h (Figure 8a). The total impedance increase was around three orders of magnitude, as also shown in the Bode magnitude plot (Figure 8e). The high impedance value is usually associated with the passive behavior of the corrosion product layer. After the second day, the phase angle shifted to a much lower frequency, suggesting slower kinetics of interfacial reactions. The change of phase angle to a more negative value may also be associated with an increase of corrosion product coverage on the surface. All the data representation showed a stable pattern at a very high resistance, indicating that the FeCO_3_ is protective between the second and seventh day.

Considering all the corrosion processes indicated by the OCP, EIS, and surface analyses data, equivalent electrical circuits are suggested for different temperatures and corrosion stages as inserted in Figure 7a,d and Figure 8a,d. R_s_ is solution resistance, Q_dl_ is a constant phase element representing double layer capacitance, and R_ct_ is charge transfer resistance. At 150 °C, additional components were added to the equivalent electrical circuit (Figure 8d). Q_c_ is a constant phase element representing the corrosion product layer capacitance and R_po_ is the corrosion product layer resistance. 

Here, Q represents constant phase element (CPE), which is assumed to have originated from the local surface distribution of reactivity. When the electrochemical reaction is coupled by an adsorbed intermediate, the non-uniform current and potential distribution may result in an increase of a low-frequency dispersion [48]. CPE parameters consist of Q and α, which are representative of a physical system. Here, capacitance is not calculated from Q and α because an interpretation of CPE impedance response to estimate either dielectric constant or film thickness in high precision requires a value for resistivity at the film-electrolyte interface, ρ_δ_ [49]. However, R_ct_, also often interpreted as corrosion resistance or polarization resistance (R_p_), was extracted to show the approximation of corrosion resistance as a function of time. Fitting result has the value of α between 0.5 and 1 with the goodness of fit (GoF) below 7.10^−4^ representing the maximum error in data fitting between 1–10%.

### 3.5. Corrosion Mechanism

The corrosion rate of carbon steel in geothermal water is influenced by the gas content, with the order of corrosivity level: O_2_ presence > CO_2_ presence > deaerated/Ar, as confirmed by the above-presented results. By comparing the specimen exposed to CO_2_ and Ar-containing solution via exposure and electrochemical tests, the corrosive effect of CO_2_ was proven to be more significant. SEM-EDX was further used to confirm the corrosion behavior of carbon steel after the test was carried out.

At 70 °C, corrosion products were dominated by Fe_2_(OH)_2_CO_3_ and FeCO_3_, whereas at 150 °C, it was dominated by only FeCO_3_ (Figure 2), which shows that FeCO_3_ formation is more favorable at 150 °C. The different morphology of corrosion products can also affect the physical and electrochemical behavior of the tested specimens, and therefore the corrosion mechanism at different temperatures. At 70 °C, although there was no significant change in E_corr_, impedance spectra of carbon steel exposed for seven days to the deaerated solution is almost 50 times higher than that of the one exposed to the CO_2_-containing solution, which evidently confirmed the corrosive effect of CO_2_.

At 150 °C, the impedance spectra of carbon steel exposed for seven days was not significantly different with or without presence of CO_2_, due to the faster formation of corrosion products in the first seven days in both conditions. However, E_corr_ showed a higher increase of carbon steel free corrosion potential when exposed to the Ar-containing solution, compared to that exposed to the CO_2_-containing solution, indicating that carbon steel is more susceptible to corrosion under CO_2_ environment. Since the seven-day exposure test was not sufficient to distinguish the corrosive effect of the CO_2_-containing solution (as shown by E_corr_) from the protective effect of FeCO_3_ layer (as shown by EIS and SEM), a longer exposure test was performed to observe the corrosion behavior and the stability of FeCO_3_ at 150 °C. Morphological observation using SEM and EDX showed that after 28 days, the FeCO_3_ structure evolved and transformed. Pitting corrosion was observed with a depth up to 72 µm in the CO_2_ presence, and 3 µm without the CO_2_ presence, which further elucidate the corrosive effect of CO_2_ at 150 °C. Additionally, there was more Ca precipitation observed after 28 days, which might also affect the stability and corrosion protection of FeCO_3_.

**Figure 9:**
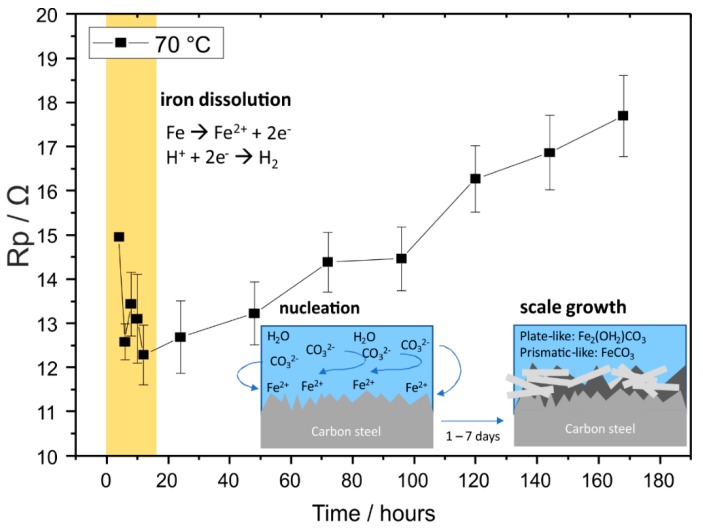
Possible corrosion mechanism of carbon steel at 70 °C in the CO_2_-containing geothermal solution and the corresponding polarisation resistance (R_p_) derived from EIS data fitting at a high–medium frequency range. Error bars indicate the error value from data fitting.

**Figure 10:**
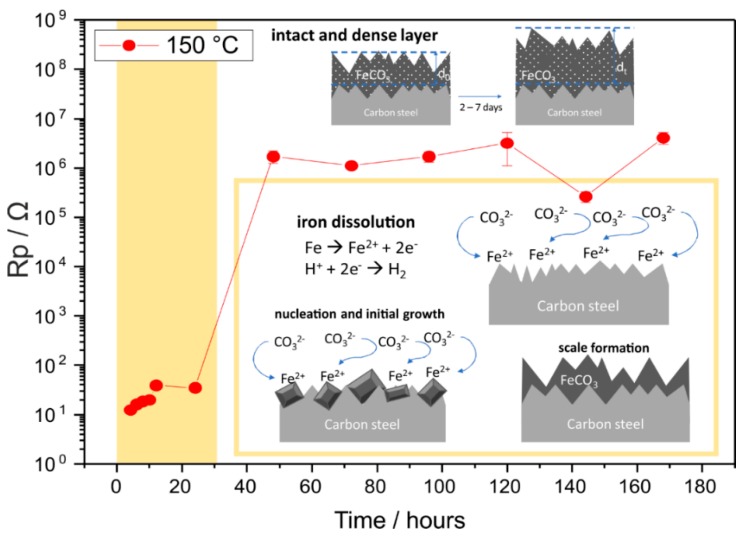
Possible corrosion mechanism of carbon steel at 150 °C in the CO_2_-containing geothermal solution and the corresponding polarisation resistance (R_p_) derived from EIS data fitting at high–medium frequency range. Error bars indicate the error value from data fitting.

It is important to note that the underlying corrosion mechanism for 70 °C and 150 °C is different, given that carbon steel remained actively corrode at 70 °C, whereas at 150 °C, there was a protective effect of FeCO_3_ seen by EIS in seven days. Therefore, the possible corrosion mechanism is proposed separately by plotting polarization resistance (R_p_) against time for results acquired at 70 °C (Figure 9) and at 150 °C (Figure 10).

At 70 °C, R_p_ decreases within the first 12 h, associated with the iron dissolution and hydrogen reduction reaction. After 12 h, R_p_ increases over time, indicating a formation of the corrosion products, i.e., Fe_2_(OH)_2_CO_3_ and FeCO_3_. All R_p_ observed until the seventh day was between 10–20 Ω and remained in the active region, suggesting a non-protective behavior of corrosion products. Although the formation and growth of FeCO_3_ and Fe_2_(OH)_2_CO_3_ was evidently shown by SEM and XRD (Figure 2a,b), pitting corrosion was also observed in the cross section (Figure 2c) with an initial pitting propagation in the area where the corrosion products are not adherent. Although there was small change in impedance spectra, it was not significant for both OCP and EIS, indicating that the main electrochemical reaction corresponds to the continuous reacting metal surface and the brine, regardless of corrosion product formation and growth.

At 150 °C, R_p_ remains below 100 Ω, which is considered stable in the active region until 24 h. There was a steep increase of R_p_ between 24 and 48 h, where polarization resistance increased exponentially at about five orders of magnitude, which showed a passive behavior, possibly due to the formation of a dense FeCO_3_ layer. This result indicated a faster crystallization of FeCO_3_ at 150 °C in only two days of exposure. After seven days, the corrosion product layer yielded a dense, compact, and adherent layer of FeCO_3_ with a thickness between 9–27 µm, as proven by the SEM image of the cross section (Figure 2f). The high R_p_ remained at the same range until seven days of exposure, which suggests that the FeCO_3_ formed at 150 °C in the presence of CO_2_ can act as a physical non-conductive barrier layer. Thus, it is clear that within two days of exposure, rapid corrosion processes take place at 150 °C, but require a longer time to validate whether FeCO_3_ is protective or not. However, at 150 °C, all experiments were carried out in the autoclaves with a limitation that under such a temperature and pressure, CO_2_ gas cannot be circulated continuously. Consequently, the corrosion mechanism at 70 °C cannot be compared directly to that at 150 °C, although the corrosive effect of CO_2_ was evidently observed at both temperatures.

Given the above-discussed results, there is room for improvement, in terms of investigating other effects regarding the corrosion behavior of carbon steel in the CO_2_-containing geothermal solution, in particular, the effect of Ca^2+^ content in the solution, and the continuous supply of CO_2_ gas at high temperatures.

## 4. Conclusions

This work contributes to a deeper understanding of carbon steel corrosion under the influence of CO_2_ in a more complex solution, i.e., artificial geothermal brine, containing 1500 mg/L of Cl^−^, 20 mg/L of SO_4_^2−^, and 15 mg/L of HCO_3_^−^, with pH 4. Exposure and electrochemical tests were performed at 70 °C and 150 °C, with and without CO_2_, to elucidate the effect of temperature and CO_2_ gas on the corrosion behavior of carbon steel. The experimental results showed that at both temperatures, carbon steel is more prone to corrosion in the presence of CO_2_ than in the Ar-containing solution. Electrochemical measurements revealed that carbon steel remained actively corroded at 70 °C, whereas at 150 °C, there was a protective effect of FeCO_3_ seen by a high E_corr_ and EIS in seven days. This is due to the crystallization of the corrosion product at 150 °C, forming a protective FeCO_3_ layer. These results indicate that carbon steel is more susceptible to corrosion at the near ground surface. However, in the deeper geothermal wells where the temperature is higher, CO_2_ contributes to a scaling formation, which might provide protectiveness to a certain degree. However, a longer exposure test of 28 days in a stagnant solution revealed a reduction of FeCO_3_ thickness and a further pit growth compared to a seven-day exposure. Based on the findings on this study, the effect of CaCO_3_ incorporation into the FeCO_3_ structure on the corrosion behavior of carbon steel should be investigated. In addition, further observation with a longer exposure time and a flowing condition of CO_2_ would be useful to replicate the real condition.

## Figures and Tables

**Table 1:** Elemental composition of carbon steel St-37 based on optical emission spectroscopy.

Element	C	Mn	P	S	Si	Cu	Ni	Cr
**wt.%**	0.0697	0.3085	0.0115	0.0229	0.049	0.0789	0.0448	0.0564

**Table 2:** Chemical composition of artificial geothermal brine as prepared in the laboratory at room temperature, with pH 4.

Ion Conc.	Cl^−^	SO_4_^2−^	HCO_3_^−^	Ca^2+^	K^+^	Na^+^
**mg/L**	1500	20	15	200	250	600

## References

[1] Gupta H.K., Roy S. (2006). Geothermal Energy: An Alternative Resource for the 21st Century.

[2] Dickson M.H., Fanelli M. (2005). Geothermal Energy: Utilization and Technology.

[3] Miller R.L., Casper L., Pinchback T. (1980). Chemistry and Materials in Geothermal Systems. Geothermal Scaling and Corrosion.

[4] Ellis A.J., Mahon W.A.J. (1977). Chemistry and Geothermal Systems.

[5] Nogara J., Zarrouk S.J. (2018). Corrosion in geothermal environment: Part 1: Fluids and their impact. Renew. Sustain. Energy Rev..

[6] Mundhenk N., Huttenloch P., Sanjuan B., Kohl T., Steger H., Zorn R. (2013). Corrosion and scaling as interrelated phenomena in an operating geothermal power plant. Corros. Sci..

[7] Bäßler R., Keserović A., Sobetzki J., Klapper H.S., Dimper M. Materials Evaluation for Geothermal Applications in Different Geothermal Waters. Proceedings of the World Geothermal Congress 2015.

[8] Nogara J., Zarrouk S.J. (2018). Corrosion in geothermal environment Part 2: Metals and alloys. Renew. Sustain. Energy Rev..

[9] Vallejo Vitaller A., Angst U.M., Elsener B. (2019). Corrosion Behaviour of L80 Steel Grade in Geothermal Power Plants in Switzerland. Metals.

[10] Darma S., DiPippo R. (2016). Indonesia: Vast geothermal potential, modest but growing exploitation. Geothermal Power Generation.

[11] Hochstein M.P., Dickson M.H., Fanelli M. (1990). Classification and assessment of geothermal resources. Small Geothermal Resources: A Guide to Development and Utilization.

[12] Hochstein M.P., Sudarman S. Indonesian Volcanic Geothermal Systems. Proceedings of the World Geothermal Congress 2015.

[13] Atmojo J.P., Itoi R., Tanaka T., Fukuda M., Sudarman S., Widiyarso A. Modeling studies of Sibayak geothermal reservoir, Northern Sumatra, Indonesia. Proceedings of the World Geothermal Congress 2000.

[14] Armstead H.C.H.E. (1973). Geothermal Energy. Review of Research and Development.

[15] Callister W.D., Rethwisch D.G. (2014). Materials Science and Engineering: An Introduction.

[16] Klapper H.S., Bäßler R., Sobetzki J., Weidauer K., Stürzbecher D. (2013). Corrosion resistance of different steel grades in the geothermal fluid of Molasse Basin. Mater. Corros..

[17] Keserović A., Bäßler R. Geothermal Systems of Indonesia—Influence of Different Factors on the Corrosion Performance of Carbon Steel API Q125. Proceedings of the World Geothermal Congress 2015.

[18] Banaś J., Lelek-Borkowska U., Mazurkiewicz B., Solarski W. (2007). Effect of CO_2_ and H_2_S on the composition and stability of passive film on iron alloys in geothermal water. Electrochim. Acta.

[19] Das Chagas Almeida T., Bandeira M.C.E., Moreira R.M., Mattos O.R. (2017). New insights on the role of CO_2_ in the mechanism of carbon steel corrosion. Corros. Sci..

[20] Kahyarian A., Brown B., Nesic S. (2017). Electrochemistry of CO_2_ corrosion of mild steel: Effect of CO_2_ on iron dissolution reaction. Corros. Sci..

[21] Farelas F., Galicia M., Brown B., Nesic S., Castaneda H. (2010). Evolution of dissolution processes at the interface of carbon steel corroding in a CO_2_ environment studied by EIS. Corros. Sci..

[22] Tanupabrungsun T., Brown B., Nesic S. Effect of pH on CO_2_ corrosion of mild steel at elevated temperatures. Proceedings of the CORROSION 2013.

[23] Mundhenk N., Huttenloch P., Bäßler R., Kohl T., Steger H., Zorn R. (2014). Electrochemical study of the corrosion of different alloys exposed to deaerated 80 °C geothermal brines containing CO_2_. Corros. Sci..

[24] Wright R.F., Brand E.R., Ziomek-Moroz M., Tylczak J.H., Ohodnicki P.R. (2018). Effect of HCO_3_^−^ on electrochemical kinetics of carbon steel corrosion in CO_2_-saturated brines. Electrochim. Acta.

[25] Nešić S. (2007). Key issues related to modelling of internal corrosion of oil and gas pipelines–A review. Corros. Sci..

[26] Al-Hassan S., Mishra B., Olson D., Salama M. (1998). Effect of microstructure on corrosion of steels in aqueous solutions containing carbon dioxide. Corrosion.

[27] Tanupabrungsun T., Young D., Brown B., Nešic S. Construction and verification of pourbaix diagrams for CO_2_ corrosion of mild steel valid up to 250 C. Proceedings of the CORROSION 2012.

[28] Mansoori H., Young D., Brown B., Nesic S., Singer M. Effect of CaCO_3_-Saturated Aqueous Solutions on CO_2_ Corrosion of Carbon Steel. Proceedings of the ECS Meeting Abstracts; 233rd ECS Meeting.

[29] Videm K., Dugstad A. (1989). Corrosion of carbon steel in an aqueous carbon dioxide environment Part 1. Mater. Perform..

[30] (2003). Standard Practice for Preparing, Cleaning, and Evaluating Corrosion Test Specimens.

[31] Soylemezoglu S., Harper R.J.G. (1982). Oxygen ingress into geothermal steam and its effect on corrosion of low carbon steel at Broadlands, New Zealand. Geothermics.

[32] Ellis P.F. (1985). Companion Study Guide to Short Course on Geothermal Corrosion and Mitigation in Low Temperature Geothermal Heating Systems.

[33] Iberl P., Alt N.S.A., Schluecker E. (2015). Evaluation of corrosion of materials for application in geothermal systems in Central Europe. Mater. Corros..

[34] Richter S., Hilbert L.R., Thorarinsdottir R. (2006). On-line corrosion monitoring in geothermal district heating systems. I. General corrosion rates. Corros. Sci..

[35] Richter S., Thorarinsdottir R., Jonsdottir F. (2007). On-line corrosion monitoring in geothermal district heating systems. II. Localized corrosion. Corros. Sci..

[36] Yang Y., Akid R. (2015). Electrochemical investigation of the corrosion of different microstructural phases of X65 pipeline steel under saturated carbon dioxide conditions. Materials.

[37] Ruhl A.S., Kotré C., Gernert U., Jekel M. (2011). Identification, quantification and localization of secondary minerals in mixed FeO fixed bed reactors. Chem. Eng. J..

[38] Pandarinathan V., Lepková K., Van Bronswijk W. (2014). Chukanovite (Fe_2_(OH)_2_CO_3_) identified as a corrosion product at sand-deposited carbon steel in CO_2_-saturated brine. Corros. Sci..

[39] Yin Z.F., Feng Y., Zhao W., Bai Z., Lin G. (2009). Effect of temperature on CO_2_ corrosion of carbon steel. Surf. Interface Anal..

[40] Mansoori H., Young D., Brown B., Singer M. (2018). Engineering. Influence of calcium and magnesium ions on CO_2_ corrosion of carbon steel in oil and gas production systems-a review. J. Nat. Gas Sci. Eng..

[41] Esmaeely S.N., Young D., Brown B., Nešić S. (2016). Effect of incorporation of calcium into iron carbonate protective layers in CO_2_ corrosion of mild steel. CORROSION.

[42] Cornell R.M., Schwertmann U. (2003). The Iron Oxides: Structure, Properties, Reactions, Occurrences and Uses.

[43] Le Hoa Q., Bäßler R., Bettge D. (2019). On the corrosion mechanism of CO_2_ transport pipeline steel caused by condensate: Synergistic effects of NO_2_ and SO_2_. Materials.

[44] Itagaki M., Taya A., Watanabe K., Noda K. (2002). Deviations of capacitive and inductive loops in the electrochemical impedance of a dissolving iron electrode. Anal. Sci..

[45] Epelboin I., Keddam M. (1970). Faradaic Impedances: Diffusion Impedance and Reaction Impedance. J. Electrochem. Soc..

[46] Barcia O.E., Mattos O.R. (1990). The role of chloride and sulphate anions in the iron dissolution mechanism studied by impedance measurements. Electrochim. Acta.

[47] Bechet B., Epelboin I., Keddam M. (1977). New data from impedance measurements concerning the anodic dissolution of iron in acidic sulphuric media. J. Electroanal. Chem. Interfacial Electrochem..

[48] Wu S.-L., Orazem M.E., Tribollet B., Vivier V. (2009). Impedance of a Disk Electrode with Reactions Involving an Adsorbed Intermediate: Local and Global Analysis. J. Electrochem. Soc..

[49] Orazem M.E., Tribollet B. (2017). Electrochemical Impedance Spectroscopy.

